# Asymmetric morality: Blame is more differentiated and more extreme than praise

**DOI:** 10.1371/journal.pone.0213544

**Published:** 2019-03-12

**Authors:** Steve Guglielmo, Bertram F. Malle

**Affiliations:** 1 Department of Psychology, Macalester College, St. Paul, MN, United States of America; 2 Department of Cognitive, Linguistic, and Psychological Sciences, Brown University, Providence, RI, United States of America; Middlesex University, UNITED KINGDOM

## Abstract

Despite extensive recent investigations of moral judgments, little is known about how negative judgments like blame might differ from positive judgments like praise. Drawing on theory from both social and moral cognition, the present studies identify and test potential asymmetries in the extremity and differentiatedness of blame as compared to praise. The *amplified blame hypothesis* predicts that people will assign greater blame for negative behaviors than praise for positive behaviors. The *differentiated blame hypothesis* predicts that, as compared to praise judgments, blame judgments will more finely differentiate among distinct mental states that precede action, such as thoughts, desires, and intentions. A series of studies—using varied stimulus sets and samples—together provide robust support for the differentiated blame hypothesis and somewhat weaker support for the amplified blame hypotheses. These results illustrate systematic asymmetries between blame and praise, generally revealing that blame is more extreme and differentiated than praise. Together, the findings reflect the social costs and social regulatory function of moral judgments, suggesting that blame and praise are not mirror images and that blame might be more complex.

## Introduction

Morality regulates social behavior by way of norms [1,2]. Norms reflect community demands on individual behavior [3] and are enforced by community approval and disapproval [4]. Some norms prohibit negative behavior, and a person violating them may be blamed; other norms prescribe positive behavior, and a person abiding by them may be praised. But are blame and praise mirror images of each other? If not, how do they differ? Previous research offers scarce evidence on asymmetries between moral judgments of blame and praise. This article takes a first step toward a systematic investigation of such potential asymmetries.

## Blame and praise

Blame has been studied extensively in the moral judgment literature, with the goal of clarifying the information elements that elicit blame, the psychological processes that generate these judgments, and the social consequences of blaming. This work has revealed that blame integrates information about outcomes and about mental states such as desires and intentions [5–7], that blame is both an intrapersonal cognitive judgment and an interpersonal social expression [8], and that blame and punishment—if applied judiciously—can help elicit cooperative, prosocial behavior [9,10].

Praise, in contrast, has not been studied extensively. Research in educational contexts has examined the conditions under which praise of performance or ability affects students’ motivation and achievement [11]. However, the information processing antecedents of praise judgments themselves have remained opaque. Moreover, comparisons of praise and blame in achievement contexts confound the valence of outcomes (success vs. failure) with the intentionality of outcomes (most of the time, success is intentional and failure is unintentional). Finally, even though the achievement context is evaluative, it is rarely *moral*, in that it pertains to pursuits of success or competence, rather than the upholding of social norms. Research on praise in educational contexts, although undoubtedly influential for policy development [12], therefore tells us little about praise as a moral judgment.

How, then, might moral judgments of blame and praise compare to each other? One possibility is that these judgments are mirror images—that they target behaviors of opposite valence but otherwise have the same underlying elicitors and information processing structure. Recent work in person perception reveals that, in addition to longstanding dimensions of warmth and competence [13,14], people also perceive others along a bipolar dimension of moral character [15–17]. Thus, positive and negative attributes of moral character are perceived as opposing ends of a single continuum or dimension. If blame and praise judgments follow this same pattern, then they, too, might be mere opposites.

However, other findings suggest that blame and praise are not mirror images but instead differ in more fundamental ways. For certain categories of behaviors, negative versions elicit substantial blame but the corresponding positive versions elicit minimal praise. Knowingly *allowing* a negative outcome to occur (despite not *intending* it) elicits strong blame, whereas knowingly allowing a positive outcome to occur elicits hardly any praise [18,19]. Further, individual differences in the tendency to blame or praise others do not appear to be opposing ends of a single continuum. Such dispositions toward blaming and praising have been shown to be orthogonal: one’s inclination to condemn morally negative behavior is wholly independent of one’s inclination to praise morally positive behavior [20].

If they are not mere opposites, what systematic differences might exist between blame and praise? Drawing upon previous research—including the small subset that has specifically compared blame to praise—we identify two hypotheses concerning candidate asymmetries between blame and praise. In particular, these hypotheses make predictions about relative differences in the extremity and differentiatedness of blame versus praise judgments.

### Amplified blame

Decades of research convincingly demonstrate that negative stimuli exert greater psychological influence than positive stimuli. Research has consistently shown that when people form moral perceptions of others, these impressions are influenced far more by negative personality characteristics than by positive ones [21,22]. Scholars have therefore argued that, across a host of different domains, negative events or stimuli are “stronger” [23] or have more “potency” [24] than corresponding positive ones. Although these accounts have rarely been applied to contexts of moral judgment, some findings reveal that people exhibit the same moral behavior when urged to be good as when urged to avoid being bad [25], perhaps suggesting that bad is no stronger than good in the moral domain. Other evidence, however, suggests such a difference. In specific instances when an identical decision happens to produce negative versus positive consequences, people typically blame the former more than they praise the latter [26–27]. We extend such accounts by assessing the prediction that, more generally, blame will be more extreme than praise. That is, the *amplified blame hypothesis* posits that people will assign more blame for negative behavior than praise for positive behavior, even when the behaviors are equated for their basic extremity (i.e., negativity/positivity).

### Differentiated blame

Beyond positing that negative events are more potent than positive ones, Rozin and Royzman [24] further argued that responses to negative events show more differentiation. Negative emotions, for example, have a greater number of elicitors and distinct labels than do positive emotions, and negative events are more fully represented in language (i.e., with a broader set of linguistic descriptors) than are positive events. This is also true for *mens rea* terms in the law and everyday life, where such descriptors as *knowingly*, *negligent*, *reckless* are applied to differentiate among negative behaviors but do not have positive counterparts.

Although previous work has not directly examined whether or how such differentiation might manifest in patterns of moral judgment, some findings suggest such a possibility. People more strongly distinguish between actions and omissions—that is, they show a stronger action-omission effect—when assigning blame than when assigning praise [28]. As compared to praise judgments for a positive act, blame judgments for a negative act are more strongly predicted by perceptions of the agent’s desire for the action [29]. Further, Pizarro, Uhlmann, and Salovey [30] showed that people blamed agents less for negative impulsive actions than for negative deliberate actions, but they praised agents as much for positive impulsive actions as for positive deliberate actions. Their additional findings revealed that, as compared to a deliberate action, people see an agent’s impulsive negative action as revealing a weaker mental commitment to the caused outcome (the agent “embraces” it less), whereas they see impulsive positive actions as revealing no less of a mental commitment to the caused outcome. These results suggest a *differentiated blame hypothesis*, which posits that people who assign blame will more finely differentiate among the agent’s degrees of mental commitment (to bringing about an action or outcome) than people who assign praise.

Can the notion of mental commitment be sharpened? On theoretical and empirical grounds, Malle and Knobe [31] suggested that *intentions* (deciding, choosing, planning to do something) come with a stronger commitment than *desires* (wanting, wishing to do something) and that intentions are the output of a deliberation process whereas desires are not. The deliberate actions in Pizarro et al.’s [30] studies therefore reflect the stronger commitment of an *intention* whereas the impulsive actions in those studies reflect the relatively weaker commitment of a *desire*. Weaker yet than desires are mere *thoughts* about a possible action or outcome—which encompass merely the consideration of its possibility, the weighing of its potential desirability. Thus, we can reformulate a sharpened *differentiated blame hypothesis*, which predicts that, across three levels of mental commitment (thought < desire < intention), blame judgments will show finer differentiation (i.e., will distinctly increase with increasing commitment) than praise judgments.

## Overview and predictions

We present a series of studies designed to test two potential asymmetries between moral judgments of blame versus praise. The *amplified blame hypothesis* predicts that, even when matched on their overall basic extremity, negative behaviors will elicit more blame than positive behaviors will elicit praise. We test this hypothesis in Studies 1, 2, 3, and 4. The *differentiated blame hypothesis* predicts that people’s blame judgments, compared with praise judgments, will more finely differentiate among distinct levels of commitment to bringing about an action or outcome. We test this hypothesis in three studies, first with a smaller set of such levels (thinking and intending: Study 1) and then an expanded set (thinking, wanting, and planning: Studies 2 and 3). To ensure that the results are replicable across a diverse set of characteristics, our studies use a variety of stimulus sets, participant samples, and judgment contexts (i.e., varying the between- vs. within-subjects manipulations of valence and judgment type).

We report all manipulations and variables, and all stimuli, data, and analysis scripts are publicly available at https://osf.io/496sv/. Across all studies, we aimed to obtain samples of at least *n* = 60 and at least *n* = 50 for all between- and within-subjects manipulations, respectively. Most samples exceeded these minimums substantially, and we aimed for larger minimum sample sizes in Study 4 (*n* = 125), which was collected online.

## Study 1

### Method

We constructed a set of 10 behavior statements (five negative and five positive), describing various behaviors that an agent might perform. For example, one negative behavior statement was “smashing the rear window of a random parked car” and one positive behavior statement was “participating in an effort to clean up a city park.” See Table A in S1 File for the complete set of behavior statements.

One hundred eighty-two undergraduate students completed a one-page questionnaire as part of a larger computer-presented survey in exchange for course credit. *Action stage* was manipulated between subjects: participants evaluated one of two mental states—one close to action completion (*intentions*) or one further away (*thoughts*)—or they evaluated completed *actions*. This latter condition served a baseline to assess whether pre-action mental states elicit weaker moral judgments than completed actions. *Valence* was manipulated within subjects: all participants rated the same five negative and five positive behavior statements (in a fixed order that alternated between positive and negative items).

Participants answered three questions, in a fixed order, about each item: *blame/praise* (“How much blame or praise would someone deserve if the person thought about [behavior statement] / intended to [behavior statement] / [behavior statement]”), *likelihood of performing* (asked only in the thinking and intending conditions: “How likely is it that the person would actually [behavior statement]”), and *basic extremity* (“Overall, how socially negative or positive is it for a person to [behavior statement]”). The blame/praise and basic extremity questions were answered on a -5 (a lot of blame/very negative) to +5 (a lot of praise/very positive) scale, and the likelihood question on a 0 (very unlikely) to 6 (very likely) scale. We then reversed the sign of blame ratings and extremity ratings for all negative items so that blame and praise ratings, as well as extremity ratings, were on a commensurable scale across valence.

### Results

**Fig 1:**
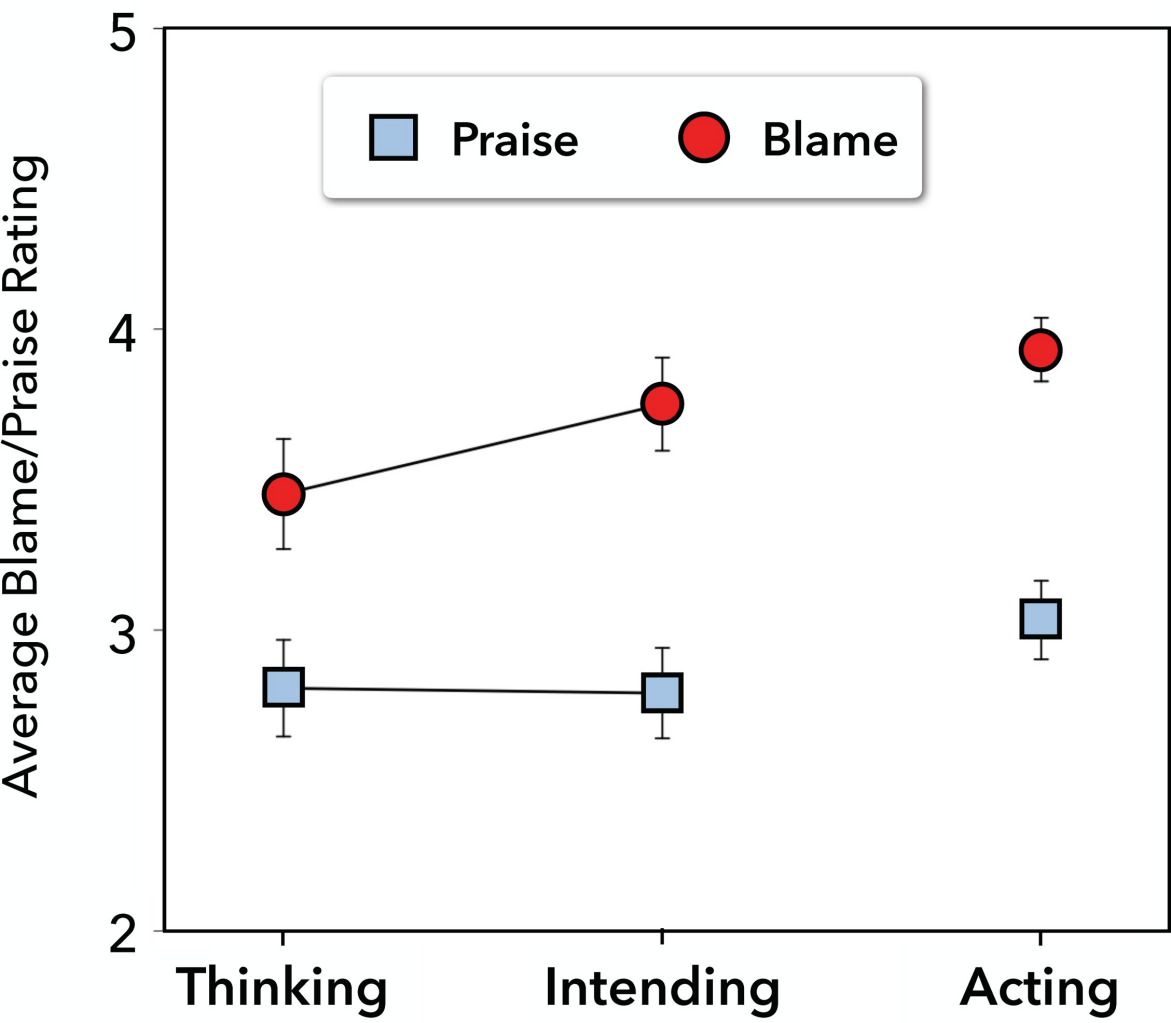
Average blame and praise ratings (±1 SE) across action stage in Study 1.

We specified a mixed-effects model, predicting trial-level praise/blame judgments from valence, action stage, and their interaction, including basic extremity (negativity/positivity) as a covariate and random intercepts for subjects. R syntax: lmer(moral~valence*cond+extremity+(1|subj), contrasts = list(cond = contr.helmert(3))). The effect of valence, controlling for basic extremity, tested the amplified blame hypothesis, and blame ratings were indeed higher (*M* = 3.71, *SD* = 1.57) than praise ratings (*M* = 2.89, *SD* = 1.48), *t*(1625) = 15.42, *p* < .001, *d* = 0.70 (see Fig 1), supporting blame amplification. The interaction contrast of thinking vs. intending by valence tested the differentiated blame hypothesis, and it too received support, *t*(1625) = 2.28, *p* = .023, *d* = 0.23, indicating that people’s differentiation between intending and thinking was stronger for blame (*M* = 3.75, *SD* = 1.58 and *M* = 3.45, *SD* = 1.73, respectively) than for praise (*M* = 2.79, *SD* = 1.50 and *M* = 2.81, *SD* = 1.56, respectively). (Moral judgments across valence were marginally stronger for acting than for the average of intending and thinking, *t*(223) = 1.71, *p* = .09.).

A similar mixed-effects model of basic extremity ratings revealed a small, though significant, valence difference: the negative items were slightly more negative (*M* = 3.59, *SD* = 1.59) than the positive items were positive (*M* = 3.43, *SD* = 1.56), *t*(1628) = 2.49, *p* = .01, *d* = 0.14. However, this difference was due to a single outlying positive item that was seen as much less positive (*M* = 2.22) than the others. We therefore excluded this single item and re-ran the preceding two models, again examining both basic extremity and praise/blame as a function of valence. With this single item excluded, the basic extremity difference was reversed: now the negative items were slightly less negative (*M* = 3.59, *SD* = 1.59) than the positive items were positive (*M* = 3.74, *SD* = 1.37), *t*(1447) = -2.66, *p* < .01, *d* = -0.14. Nonetheless, the amplified blame pattern remained robust: controlling for basic extremity, blame ratings were again substantially higher (*M* = 3.71, *SD* = 1.57) than praise ratings (*M* = 3.10, *SD* = 1.43), *t*(1447) = 12.79, *p* < .001, *d* = 0.50.

Lastly, a similar mixed-effects model examining likelihood judgments for the thinking and intending cases (likelihood for acting was meaningless and therefore not probed) revealed that negative thoughts and intentions were actually less likely to be acted upon (*M* = 2.62, *SD* = 1.62) than positive thoughts and intentions (*M* = 3.83, *SD* = 1.42), *t*(1097) = 15.1, *p* < .001, *d* = 1.20. Thus, the finding that blame was more extreme than praise is not due to an inference that negative thoughts or intentions somehow more easily come to fruition than positive ones.

### Discussion

The results of Study 1 revealed support for the amplified and differentiated blame hypotheses. Consistent with the amplified blame hypothesis, while holding constant the basic extremity of the items (as a covariate in the model), people assigned more blame for negative behaviors than praise for positive behaviors. Consistent with the differentiated blame hypothesis, people more finely differentiated between thoughts and intentions when assigning blame than when assigning praise. This pattern is particularly noteworthy given that action stage was manipulated between subjects; although people could not directly compare different stages to one another, they still systematically differentiated between them. Nonetheless, the between-subjects nature of this manipulation might provide too little statistical power to adequately test the differentiation hypothesis, and this lack of power might also explain why, surprisingly, moral judgments were only marginally stronger for actions than for mental states (thoughts and intentions combined). Moreover, even though the amplification effect emerged while statistically controlling for basic extremity, we were not perfectly successful in equating this dimension across valence: the negative items were seen as slightly more negative than the positive items were seen as positive. We address this concern and make additional improvements in our next study.

In Study 2 we sought to replicate the patterns of Study 1 while extending the methodology in several important ways. First, to ensure that the findings generalize to a context in which participants simultaneously evaluate multiple action stages, we manipulated action stage within subjects. Second, to ensure that the findings generalize to a wide array of mental states, we varied and expanded the set of mental states that people evaluated. In particular, we replaced *intending* with the conceptually similar [31] term *planning*, which is twice as common in ordinary English [32]. We also included an additional state—*wanting—*that is conceptually identical to *desires* (but more colloquially typical) and is intermediate between *thinking* and *planning*, thus enabling a more fine-grained test of the differentiated blame hypothesis. Third, to ensure an even more precise matching of items on their basic extremity, we preselected items based on their pretested ratings on this dimension, rather than merely evaluating such differences in a posttest. Finally, to ensure that the findings hold regardless of whether participants evaluate negative and positive behaviors together or separately, we had some participants rate items of a single valence, while other participants rated both negative and positive behaviors.

## Study 2

### Method

Before proceeding with the main study, we obtained ratings of basic extremity and selected a matched set of behaviors accordingly. Some behaviors were selected based on their average rating (-5 to +5) from Study 1. Some were selected from Fuhrman, Bodenhausen, and Lichtenstein [33], who had participants rate the extremity of various behavior statements using a slightly different 11-point scale (0 = “extremely bad” to 10 = “extremely good”). We also generated additional behavior statements and obtained corresponding ratings. In one instance, we instead used a 9-point scale (-4 to +4); we converted these ratings to a -5 to +5 scale (original rating * 5/4), and we did the same for the Furhman et al. [33] ratings (original rating—5). We then selected a final set of eight negative (*M* = -3.00) and eight positive behaviors (*M* = 2.99), such that the two subsets were equivalent in overall basic extremity, and, moreover, such that each behavior had an opposite-valence counterpart with a near-identical rating. As one example, the negativity of the most extreme negative behavior (“set fire to his house to get insurance money for it”) and positivity of the most extreme positive behavior (“paid a month’s rent for a family threatened to be evicted”) were perfectly matched (*M* = -4.53 and *M* = 4.53, respectively). See Table B in S1 File for the complete set of eight negative and eight positive behaviors and their pretested ratings.

Ninety-two adults completed the study while waiting at a public transit center. Each rated 16 items, comprised of four unique behaviors at each of four action stages: *thinking* (“A person thought about [behavior statement]”); *wanting* (“A person wanted to [behavior statement]”); *planning* (“A person planned to [behavior statement]”); and *acting* (“A person [behavior statement]”). Thus, action stage was manipulated within subjects. Valence was manipulated in both a within- and between-subjects manner. Participants in the *dual valence* sample (*n* = 42) rated both negative and positive items (eight of each), whereas participants in the *single valence* sample rated 16 negative (*n* = 26) items or 16 positive (*n* = 24) items. To vary the order of presentation, we used eight distinct item orders (two negative-only, two positive-only, four dual valence). Mirroring the structure of the full set of 16 behaviors, we constructed these item orders such that the negative and positive behaviors again had near-identical basic extremity ratings. For example, within each of the four dual valence orders, the mean basic negativity of the negative items differed from the mean basic positivity of the positive items by .07 or less.

For each negative behavior, participants responded on a unipolar blame scale, ranging from 0 (none at all) to 7 (maximum possible) scale; for each positive behavior, they responded on a unipolar praise scale, ranging from 0 (none at all) to 7 (maximum possible).

### Results

We first examined whether moral judgment patterns across the four action stages differed between the single valence and dual valence conditions. Two mixed-effect models—one for each moral judgment type—revealed that, for judgments of both blame and praise, there was no significant *action stage* × *valence composition* (single vs. dual valence) interaction, both *F*s < 1.25. Thus, any effects of action stage on blame and praise were consistent regardless of whether people evaluated behaviors of a single valence or of both valences. The valence composition variable was therefore omitted from all subsequent analyses. In addition to the models below, which exclude valence composition, we also specified models that included valence composition as a covariate. In every case, each effect yielded the same conclusion with respect to statistical (non) significance, regardless of whether valence composition was included or excluded as a covariate.

We then assessed our primary hypotheses. Since our items were now appropriately equated on basic extremity across valence (e.g., the most extreme negative and positive items were designated as item 1), we now included random intercepts for items as well. Thus, we tested the amplified blame hypothesis with a mixed-effects model predicting item-level moral judgment ratings by valence, including random intercepts for subjects and for items. R syntax: lmer(rating~valence+(1|subj)+(1|item)). Surprisingly, blame ratings were no higher (*M* = 4.21, *SD* = 2.32) than praise ratings (*M* = 3.99, *SD* = 2.32), *t* = .20, *p* = .85, *d*s = .34 (between-subjects comparison in the single valence conditions) and -.06 (within-subjects comparison in the dual valence condition), counter to the blame amplification prediction.

We then conducted two separate mixed-effect models—one for each valence—to examine how blame and praise differed as a function of action stage. We specified three contrasts on the action stage variable. The first contrasts compared completed actions to pre-action mental states (the average of thinking, wanting, and planning). The subsequent two contrasts examined polynomial patterns—linear and quadratic—across the three mental states. The key test of the differentiated blame hypothesis concerns the linear pattern across these mental states and whether it is moderated by valence. We had no particular predictions regarding quadratic effects, but we included tests of this effect both for completeness and because the clearest case for a purely linear increase in ratings would be the presence of a linear effect combined with the absence of a quadratic effect.

**Fig 2:**
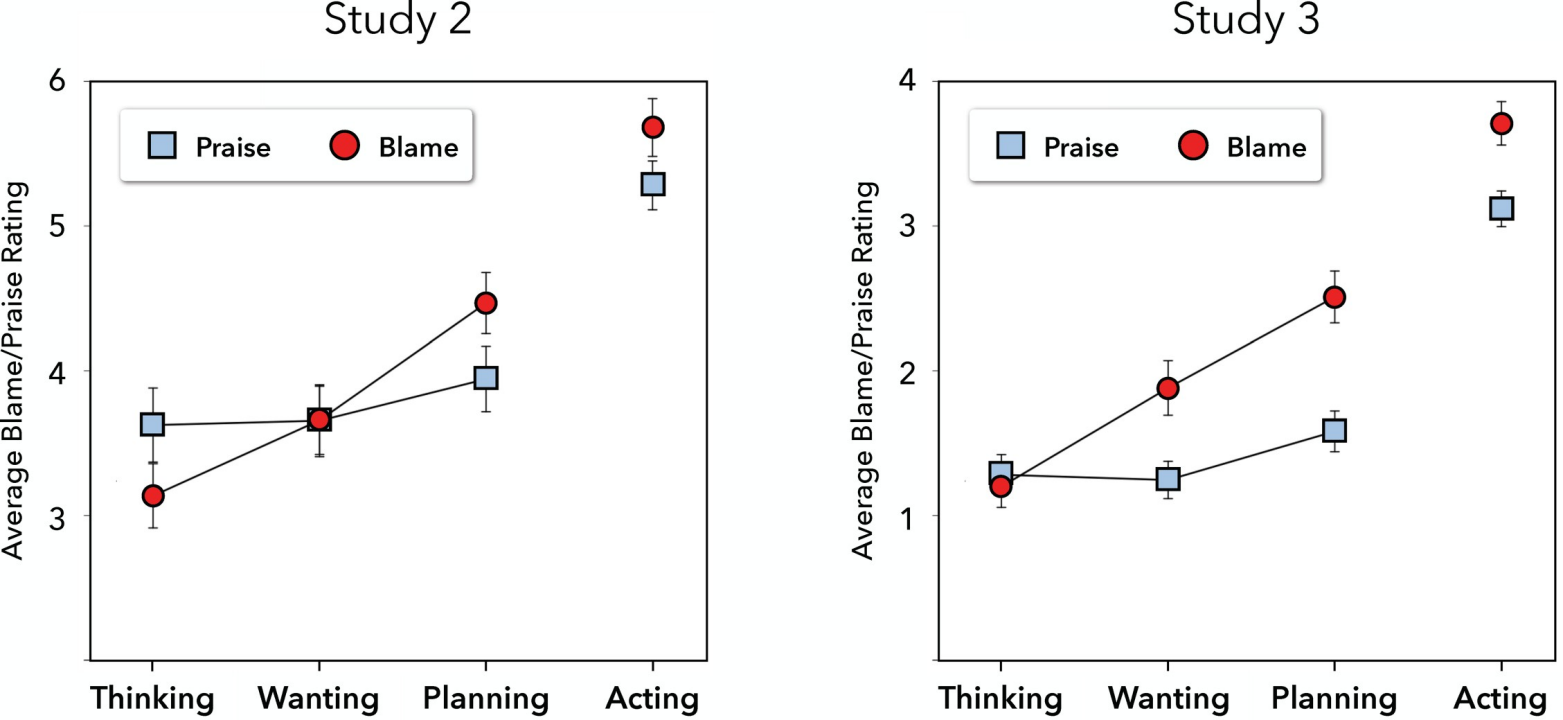
Average blame and praise ratings (±1 SE) across action stages in Study 2 and Study 3.

Among positive behaviors, actions elicited more praise than did mental states, *t*(645) = 11.3, *p* < .001, *d*s = .83 and 1.06 (between- and within-subjects comparisons, respectively). Polynomial contrasts revealed a small linear increase in praise ratings from thinking (*M* = 3.47, *SD* = 2.39) to wanting (*M* = 3.52, *SD* = 2.33) to planning (*M* = 3.87, *SD* = 2.18), *t*(645) = 2.43, *p* = .015, *d*s = .28 and .10 (between- and within-subjects comparisons, respectively); there was no quadratic pattern to these ratings, *t* = 1.11, *p* = .26. Among negative behaviors, actions elicited more blame than did mental states, *t*(665) = 14.8, *p* < .001, *d*s = 1.67 and .96 (between- and within-subjects comparisons, respectively). There again was a linear increase in blame ratings from thinking (*M* = 3.13, *SD* = 2.16) to wanting (*M* = 3.60, *SD* = 2.32) to planning (*M* = 4.40, *SD* = 2.09), *t*(666) = 7.87, *p* < .001, *d*s = .73 and .75 (between- and within-subjects comparisons, respectively), but no quadratic pattern, *t* = 1.13, *p* = .26. Consistent with the differentiated blame hypothesis, the increase in moral judgment severity across mental states was stronger for blame than for praise: a final mixed-effects model revealed that the linear pattern was moderated by behavior valence, *t*(1357) = 3.75, *p* < .001, *d*s = .41 and .64 (between- and within-subjects comparisons, respectively). (see Fig 2).

### Discussion

Study 2 modified the methodology of Study 1 by including a different set of mental states (replacing ‘intending’ with the conceptually similar ‘planning’ and adding ‘wanting’) and by having participants evaluate all action stages rather than just a single one. The amplified blame hypothesis was not supported in this study. We found that, unsurprisingly, both praise and blame were more severe for completed actions than for any pre-action mental states. Further, the differentiated blame hypothesis received strong support. Blame was more finely differentiated among thinking about, wanting, and planning to perform a negative action than praise was for thinking about, wanting, and planning to perform a positive action.

## Study 3

Study 3 served as a replication of the patterns revealed in Study 2, focusing specifically on the dual valence (within-subject) presentation mode. We also returned to the bipolar response scale from Study 1 (-5 to +5) so that people use the same scale to judge positive and negative behaviors. This way, any differences between behavior sets cannot be due to differences in scale use.

### Method

Fifty-five adults completed the study while waiting at a public transit center. As in the dual valence condition of Study 2, each participant rated 16 items (eight negative and eight positive), comprised of four unique behaviors at each of the *thinking*; *wanting*; *planning*; and *acting* action stages. To vary the order of presentation, we used four distinct item orders.

For each behavior, participants responded on a bipolar moral judgment scale, ranging from -5 (a lot of blame) to +5 (a lot of praise).

### Results

Before conducting any analyses, we reversed the sign of the ratings for all negative items (i.e., multiplying by -1), so that the blame and praise ratings would be directly comparable. We then examined the same mixed-effects models as in Study 2 to assess the amplified and differentiated blame hypotheses. All models assessed trial-level ratings and included random intercepts for subjects and for items. The first model, including behavior valence as the sole predictor, revealed that blame was higher overall (*M* = 2.32, *SD* = 1.81) than was praise (*M* = 1.81, *SD* = 1.57), *t*(817) = 5.25, *p* < .001, *d* = 0.56, consistent with the amplified blame hypothesis (see Fig 2).

We then ran separate models for each valence, in which we specified the same contrasts as in Study 2: first comparing actions to the average of all pre-action mental states, and then examining linear and quadratic patterns across the three mental states. Among positive behaviors, actions elicited more praise than did mental states, *t*(377) = 15.7, *p* < .001, *d* = 1.94. There was a significant linear trend in praise ratings from thinking (*M* = 1.28, *SD* = 1.51) through wanting (*M* = 1.24, *SD* = 1.17) to planning (*M* = 1.58, *SD* = 1.33), *t*(377) = 2.21, *p* = .03, *d* = 0.29; the quadratic pattern did not reach statistical significance, *t* = 1.63, *p* = .10. Among negative behaviors, actions elicited more blame than did mental states, *t*(376) = 14.4, *p* < .001, *d* = 1.66. Likewise, there was a significant linear trend in blame ratings from thinking (*M* = 1.20, *SD* = 1.24) through wanting (*M* = 1.88, *SD* = 1.56) to planning (*M* = 2.51, *SD* = 1.81), *t*(376) = 8.32, *p* < .001, *d* = 1.08; there was no quadratic pattern to these ratings, *t* = .10. As in Study 2, and consistent with the differentiated blame hypothesis, the increase in moral judgment severity across pre-action stages was again stronger for blame than for praise: a final mixed-effects model revealed that the linear pattern was moderated by behavior valence, *t*(811) = 4.49, *p* < .001, *d* = 0.77.

**Fig 3:**
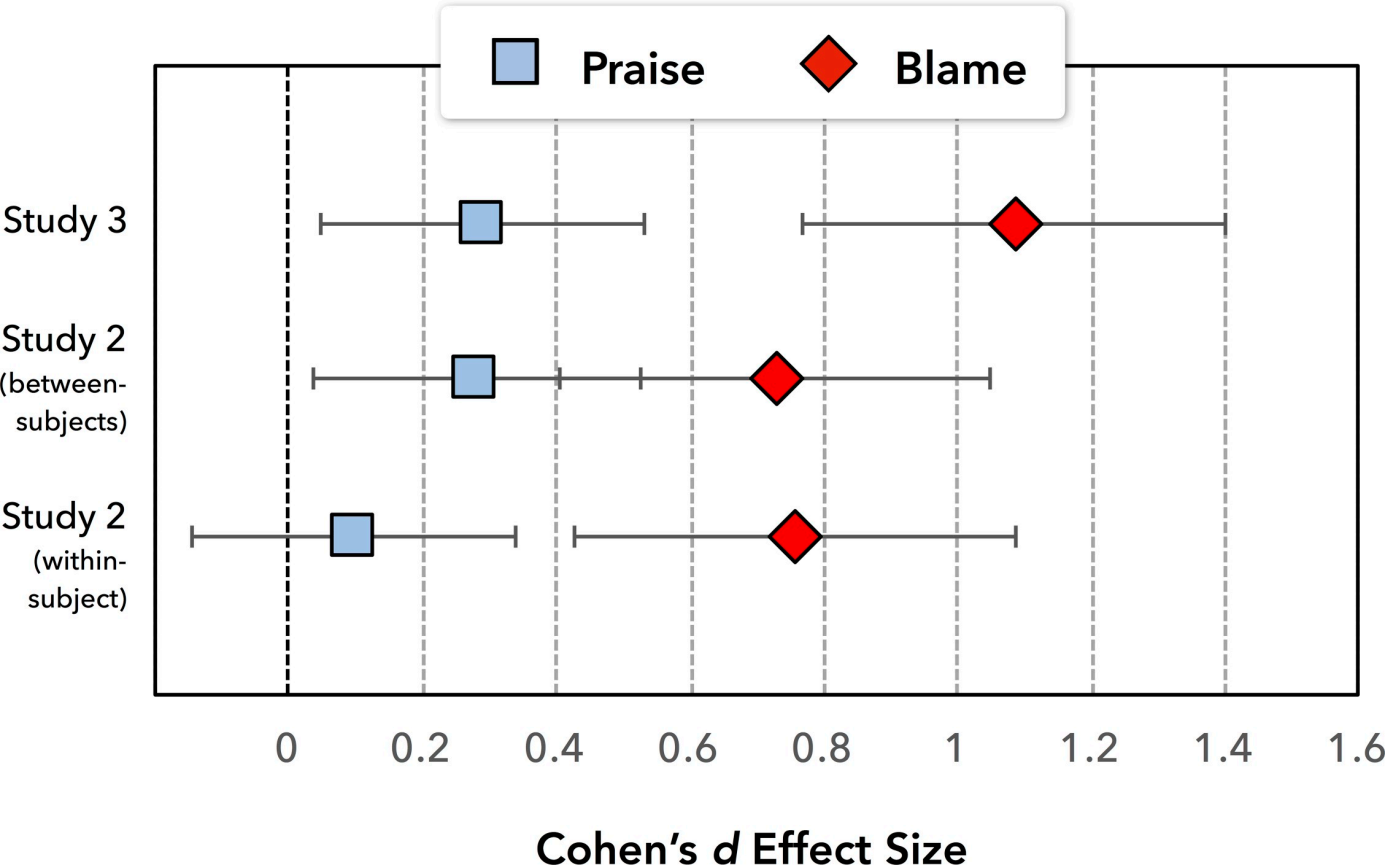
Meta-analysis of the differentiation effect for praise and blame. Depicts effect sizes for the differentiation effect—the linear increase in ratings from thinking through wanting to planning—for praise (blue) and blame (red) across Studies 2 and 3 (including separate between- and within-subjects subsamples in Study 2).

To examine the consistency of the blame-praise differentiation effect we performed a meta-analysis on three samples (Study 3 and separate between- and within-subject subsamples in Study 2). Fig 3 displays the linear contrast effect sizes for blame and praise separately, but we computed the random-effects average on the interaction term, yielding $\bar{d}$ = 0.671, 95% CI [0.442; 0.899], *z* = 5.76, *p* < .001. (Details on the calculation of effect sizes and their variances can be found in the Supporting Information.)

## Discussion

Studies 1, 2, and 3 assessed two hypotheses. According to the amplified blame hypothesis, people’s blame judgments are more extreme than their praise judgments, even when the negative and positive behaviors are matched on their extremity. According to the differentiated blame hypothesis, people more finely differentiate among distinct pre-action mental states when assigning blame than when assigning praise. Evidence for blame amplification was somewhat inconsistent—the pattern of means was present in all three studies, but whereas Studies 1 and 3 showed this pattern to be statistically significant, Study 2 did not.

Evidence for the differentiated blame hypothesis was consistent. In all three studies, people more finely differentiated among different mental states (thinking about, wanting, or planning/intending to perform an action) when assigning blame than when assigning praise. These patterns held true across several methodological variations: different sets of mental states; between- and within-subject manipulations of action stage and valence; and different response scales for assessing blame and praise.

We conducted a follow-up investigation to take a step toward accounting for the differentiation asymmetry. We used the stimuli from Study 3 and examined the thinking, wanting, and planning action stages. Participants (*N* = 263 from MTurk) rated either *commitment* (“How committed do you think the person is to completing the described action?”) or *likelihood* (“How likely do you think it is that the person will complete the described action?”), providing ratings for each of the three action stages, for each of four positive and four negative behaviors, for a total of 24 distinct ratings. In one additional condition that we don’t report here, we asked about *typicality* (“How common do you think it is for someone to think about/want/plan to do this?”).

There was a main effect of valence for each variable: agents with negative mental states were overall perceived as less committed and less likely to act than those with positive mental states, both *t*s > 9.30. More importantly, within each valence, perceived commitment and likelihood increased in a linear fashion from the thinking to wanting to planning action stages, all *t*s > 9.90, thus confirming that people perceive planning as being “closest” to action completion and merely thinking to be furthest away. In contrast to the moral judgment findings in Studies 2 and 3, however, the linear patterns in commitment and likelihood ratings were not moderated by valence, both *t*s < .15. Thus, although blame is more differentiated than praise, perceived commitment and likelihood do not show greater differentiation depending on valence.

The preceding analyses showed that people’s commitment and likelihood ratings, averaged over items, increased across pre-action states at similar rates for negative and positive behavior. We conducted one final test to determine whether the variation of these ratings across items was more closely linked to blame than to praise. To assess whether behaviors that showed greater differentiation in commitment/likelihood across action stages also showed greater differentiation in moral judgments, we examined ratings aggregated over participants for each of the individual 16 base items (eight negative and eight positive). For each item, we computed a difference score representing the change in average commitment/likelihood between the thinking and planning stage. The greater this difference score, the greater the diagnosticity of planning (relative to thinking) with respect to commitment/likelihood of acting. In a similar fashion, we used the moral judgment ratings from Study 3 to compute a difference score for each item representing the change in average blame/praise between thinking and planning. We then examined the correlation between these two sets of difference scores to determine whether items that showed greater diagnosticity differences also showed greater moral judgment differences. For negative items, this was indeed the case: behaviors for which people perceived greater differences in likelihood/commitment between thinking and planning also showed greater blame differences between thinking and planning, *r* = .52 (commitment) and *r* = .50 (likelihood). That is, when planning becomes especially diagnostic of an action, blame increases. These patterns were weaker and inconsistent for positive items. Greater differences in perceived commitment were only weakly related to greater praise differences, *r* = .24, and greater differences in perceived likelihood were related to *smaller* praise differences, *r* = -.41. (Since each correlation had *df* = 6 [computed based on the respective eight behaviors], none reached the conventional level of significance). Overall, these correlations suggest that for negative (but not positive) behavior, as one’s specific mental state becomes a clearer indicator of one’s commitment to and likelihood of acting, blame increases correspondingly. Together, then, our findings show that whereas thoughts, desires, and intentions taken as *classes* of mental states are increasingly diagnostic of action completion for both negative and positive behavior, blame more closely tracks the varying diagnosticity of specific thoughts, desires, and intentions than does praise.

## Study 4

Because there was some inconsistency in the evidence for the amplification hypothesis, Study 4 tested it one more time, with a new, tightly constructed stimulus set. In this set, the descriptions of negative and positive behaviors were not only matched on overall negativity/positivity but also on several specific content features and statement length.

### Method

#### Stimulus construction

We aimed to construct a set of sentences that would satisfy the following properties: (a) each sentence base would have negative, positive, and neutral variants; (b) across the set of all sentences, the negative and positive sentences would be equated on their basic negativity/positivity; and (c) the variants would be linguistically identical except for a key verb (or verb phrase) that differentiates them.

We created 15 sentence bases, each with negative, positive, and neutral variants. For example, one sentence base with its three valence variants was “Tracy decided to [steal from] [donate to] [read about] a children’s charity.” We then obtained pretesting ratings from participants (*N* = 152) recruited from MTurk. Each participant rated 15 sentences (five per valence; only one per sentence base), presented in a random order. They indicated “how negative or positive you think each behavior is” on a scale from -4 (very negative) to +4 (very positive).

From the resulting basic extremity ratings of this complete set of 15 sentence bases, we identified a subset of nine that satisfied the properties listed above (see Table C in S1 File). Collapsing across all nine sentences, the valence extremity (basic negativity/positivity) of the negative sentences (*M* = -2.55) was nearly identical to that of the positive sentences (*M* = 2.51), *t*(16) = .22, *p* = .83, *d* = 0.11. This was also true at the level of individual sentences: for each of the nine sentences, the yoked negative and positive variants did not differ in valence extremity, all *t*s < 1.37, all *p*s > .17, all *d*s < .28. The average extremity of the negative and positive sentences taken together (*M* = 2.53) differed dramatically from the average extremity of the neutral sentences (*M* = .50), *t*(24) = 8.90, *p* < .001, *d* = 3.63.

#### Design and procedure

We next created two versions of the same underlying study. All participants (*N* = 422) completed the study on MTurk. In both versions of the study, participants read a series of 18 sentences (three practice items plus 15 experimental items) and provided a single judgment about each one. The 15 experimental items consisted of nine sentences from the matched set described above (three negative, three positive, three neutral; exactly one sentence for each sentence base), plus a fixed set of six sentences depicting accidental behavior (e.g., “As he was leaving the car, Daryl pinched his fingers in the door.”), which were included to provide a balance of behaviors that likely would (and would not) elicit moral judgments. The pairing of each sentence base with each valence was counterbalanced across participants.

In sample A (*N* = 168), participants answered one of three judgment probes for any given sentence, and the particular probe they received was randomly selected for each sentence. Probes were always presented as single-word cues (a variation of the procedure used by Malle & Holbrook [34]), whose full meanings participants had learned in the instructions phase: BLAME? (“Does the main character deserve blame for the behavior?”); PRAISE? (“Does the main character deserve praise for the behavior?”); and INTENTIONAL? (“Was the main character’s behavior intentional?”). In sample B (*N* = 254), participants always answered the same probe (either PRAISE? or BLAME?) throughout the entirety of the experiment. Thus, judgment type was manipulated within-subjects in sample A and between-subjects in sample B. In two other variations whose results we don’t report here, the probes BAD?, GOOD?, and INTENTIONAL? were used in both a within- and between-subjects design. These probes allow for an examination of differences between moral judgments and mental state judgments, as well as differences between moral judgments and valenced (but not necessarily moral) judgments. All judgment probes were answered on a 1–7 scale—with higher values indicating stronger judgment—and participants provided their responses via keypress of the corresponding numeric button (cf. [35]). Here we restrict our focus specifically to the ratings for blame and praise.

### Results

**Fig 4:**
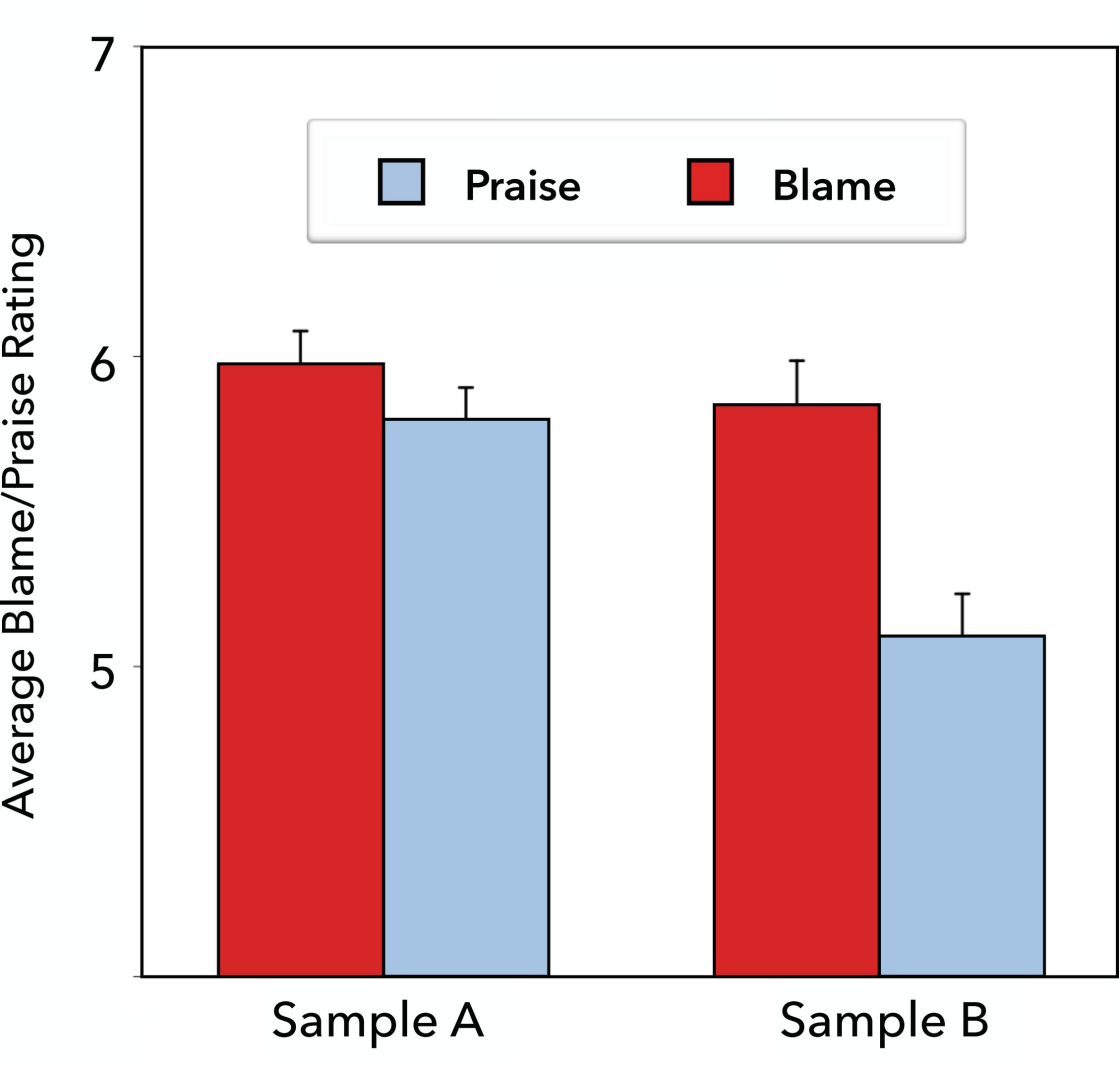
Average blame and praise ratings (+1 SE) in Study 4 Sample A (multiple question probes) and Sample B (one question probe).

In each of the two samples, we conducted a multilevel model with trials as the unit of analysis, including random intercepts for subjects. Disconfirming the amplified blame hypothesis, in Sample A (multiple question probes) there was no difference in the magnitude of blame for negative behavior (*M* = 5.97, *SD* = 1.37) versus praise for positive behavior (*M* = 5.80, *SD* = 1.38), *t*(266) = .60, *p* > .10. Since probes were selected randomly, a subset of participants rated only blame for negative behavior (*M* = 6.05) and another subset rated only praise for positive behavior (*M* = 5.60), *d* = 0.34; a third subset provided both types of ratings (*M* = 5.78 and *M* = 5.91, respectively), *d* = -0.10. In Sample B (single question probe), the amplified blame hypothesis received support, as people assigned more blame for negative behavior (*M* = 5.84, *SD* = 1.70) than praise for positive behavior (*M* = 5.10, *SD* = 1.88), *t*(160) = 3.72, *p* < .001, *d* = 0.59. The patterns in both samples are depicted in Fig 4.

### Discussion

In a carefully constructed and balanced stimulus set, we again found somewhat inconsistent evidence for the amplified blame hypothesis. Stronger blame for negative behaviors than praise for positive behaviors (matched for basic extremity) robustly emerged in sample B (in which participants made a single type of judgment throughout the experiment). By contrast, this pattern did not emerge nearly as strongly (and not significantly) in sample A (in which participants made three different types of judgments). However, a closer look at the data in sample A does provide more evidence for the hypothesis than against it. A single behavior (“While updating the office computer system…”) elicited substantially more praise (*M* = 6.47) than blame (*M* = 5.09). Among the remaining behaviors, blame was marginally higher overall (*M* = 6.10, *SD* = 1.25) than was praise (*M* = 5.72, *SD* = 1.38), *t*(253) = 1.78, *p* = .076, *d* = 0.29.

**Fig 5:**
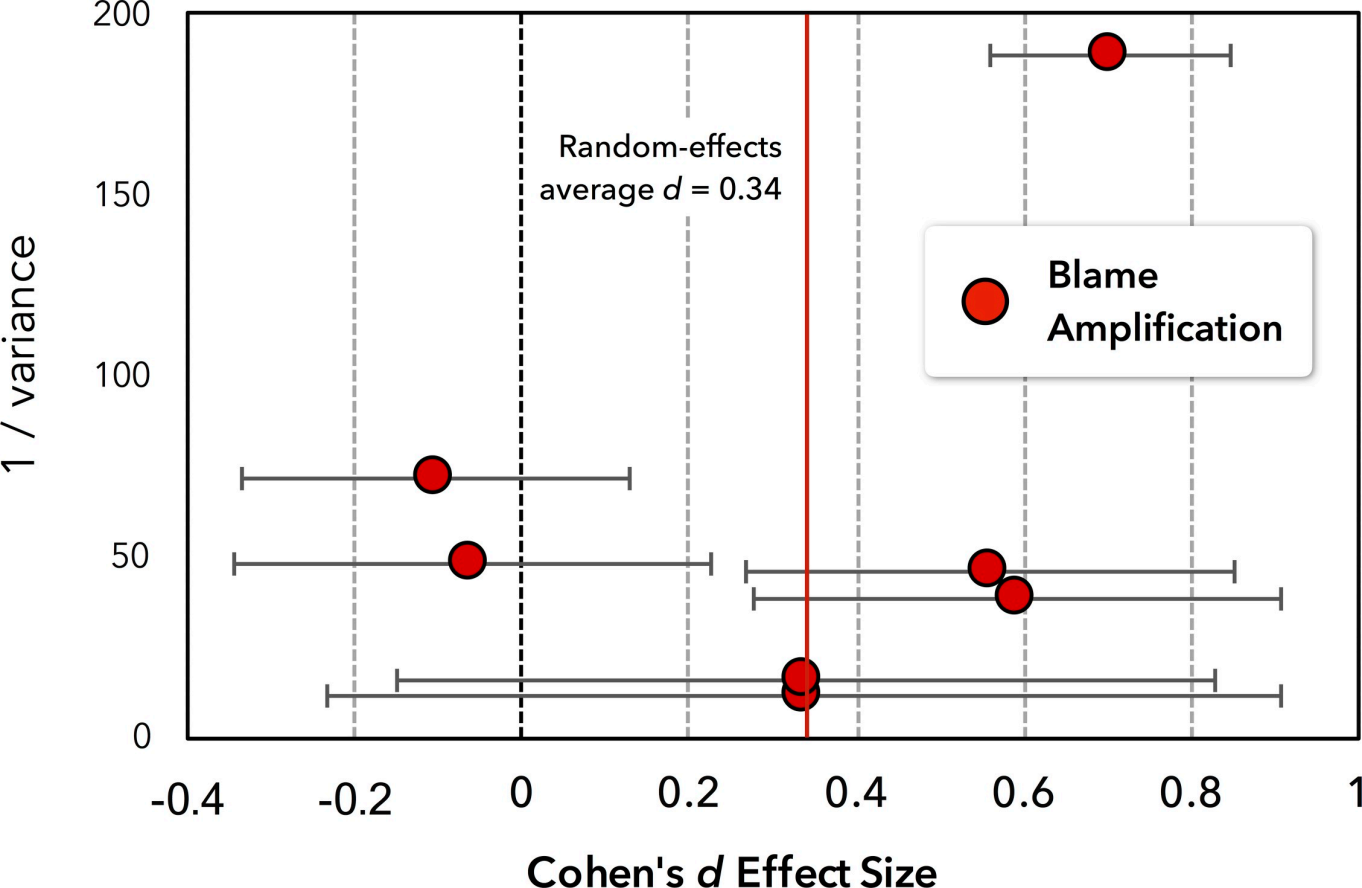
Meta-analysis of blame amplification effects from seven subsamples in Studies 1 to 4. Effects with larger weights (1/σ^2^) are based on larger sample sizes and contribute more strongly to the average effect size of d = 0.34.

The blame amplification effect showed some inconsistency across the studies, so we performed a meta-analysis on seven samples (Study 1, Study 3, Study 4B, as well as separate between- and within-subject subsamples in Studies 2 and 4A). The random-effects average (Fig 5) was $\bar{d}$ = 0.34, 95% CI [0.038; 0 .641], *z* = 2.21, *p* = .027, providing some confidence that blame amplification, though varying across samples, is a real phenomenon. (Details on the calculation of effect sizes and their variances can be found in the Supporting Information.)

## General discussion

The present studies assessed potential differences between judgments of blame and praise. Drawing upon research examining cognitive processing of negative and positive stimuli broadly speaking, we developed hypotheses concerning two ways in which blame and praise, specifically, might be asymmetric. The amplified blame hypothesis posited that people will blame negative behavior more strongly than they will praise positive behavior; the differentiated blame hypothesis posited that people will more finely distinguish among discrete mental states preceding action—such as thinking, wanting, and intending—when assigning blame than when assigning praise.

At the broadest level, our results indicate that blame and praise are not mirror images but differ in systematic ways. More specifically, our results provide partial support for the amplified blame hypothesis and consistent support for the differentiated blame hypothesis. We conducted these hypothesis tests across several methodological variations, including different stimulus sets, diverse sample types (undergraduates, community members, and online participants), and the between- vs. within-subjects manipulation of valence, action stage, and question type.

### Amplified and differentiated blame

Previous work has shown that negative events lead to stronger [23] and more differentiated [24] psychological processing than positive events. The current findings show that similar patterns emerge in the context of moral judgment, whereby blame judgments are both more amplified and differentiated than praise judgments. The amplified blame effect was the weaker of the two patterns, emerging significantly in some but not all of the tests, with an average effect size of *d* = 0.34. This suggests that amplified blame appears to be a real—though not overwhelmingly large—effect.

Evidence for the differentiated blame hypothesis was consistent and robust, with an average effect size of *d* = 0.67. Extending previous work demonstrating people’s tendency to morally evaluate mental states [36], our findings show that mental states matter in different ways for blame judgments as compared to praise judgments. Since members of any community are motivated to minimize the occurrence of other members’ negative behavior, blame is useful for proactively discouraging possible or probable negative acts. Thus, people’s blaming of a thought or plan might serve to preemptively steer a person away from committing bad acts that they were tempted to commit. However, people don’t apply blame indiscriminately to any hint of a culpable mental state. Rather, blame is applied in a graded fashion, with greater blame for mental states such as negative intentions, which are temporally close to and more diagnostic of transgressions, and less blame for mental states such as negative thoughts, which are temporally distant from and less diagnostic of transgressions.

Social perceivers face different motivations, though, when assigning praise. Since praise serves to reinforce others’ positive behavior, it will be most effective when the target has already performed the behavior one wishes to reinforce. Doling out too much praise preemptively—merely for positive thoughts or desires—might, in fact, be counterproductive, disincentivizing the target from following through on the behavior that the perceiver wanted to encourage in the first place. When assigning blame, therefore, perceivers care about *how close* a target is to acting negatively (prevention becomes more urgent), but when assigning praise, they care primarily about *whether* the target has acted positively. Targets earn a minimal degree of praise for positive (not yet acted-upon) mental states, but not in a differentiated way depending on the particular mental state.

Future research can nonetheless further explore the differentiation effect to more precisely determine its explanatory mechanism. Beyond differing in their likelihood of completion, mental states such as plans and intentions are also seen as more controllable than others such as desires and hopes [37]. Thus, people might show differentiated blame responses because mental states that are proximal to action are also indicative or greater effort and intentionality, thereby constituting more severe moral violations and eliciting greater blame. Relatedly, the differentiated blame pattern might reflect inferences about moral character, since culpable mental states are seen as evidence of poor moral character [37,38] and moral character can itself influence blame [39].

### Other potential asymmetries between blame and praise

Other blame-praise asymmetries might exist beyond those that we have reported here. Since negative information is more readily detected and attended to than positive information [23,40,41], blame is likely to be used more frequently than praise. Thus, whereas we have shown that people assign a greater amount of blame than praise, people might also assign blame more often, or across a wider range of behaviors. At a systemic level, this is precisely the approach implemented by the legal system, which is designed primarily to sanction negative behavior rather than to reward or promote positive behavior [42]. This structure is not inevitable, though, and some contexts have shown that a reversal is possible. Recent strategies in education, for example, have found success by positively reinforcing socially desirable behavior, rather than punishing socially problematic behavior [43]. Policies that adopt this type of structure—focusing on “carrots” instead of “sticks”—tend to be well received [44]. And so long as rewards can readily be allocated—especially when they lead to favorable outcomes for the collective good—people are inclined to forgo blame and punishment in favor of praise and reward, which can boost cooperation and improve outcomes for all parties involved [45]. So while blame typically dominates over praise, it is nonetheless possible to override this dominance, often to the benefit of the collective.

Further, existing theoretical and empirical accounts offer competing predictions about the relative speed of blame and praise. On the one hand, negative acts, outcomes, or metal states can elicit immediate negative spontaneous evaluations and a motivation to blame [5,46], thus leading to heightened blame-consistent perceptions, such as greater causal influence or more culpable mental states [39,47]. These findings suggest that people are highly motivated to assign blame, leading to the prediction that people will be faster to assign blame than to assign praise.

On the other hand, prosocial responses often come quickly or intuitively, including people’s tendencies to be honest [48–49] and to cooperate [50] Further, blame judgments usually require “warrant,” or a justification for why, and to what extent, blame is being assigned [8,51], and allocating blame in an unjustified or excessive manner can lead to adverse consequences, including retaliation [52] and the deterioration of social relationships [53]. These findings suggest that blame is an especially costly and consequential judgment, leading to the prediction that people will be slower to assign blame than to assign praise. We are currently conducting a series of studies to assess these competing predictions regarding the relative speed of blame versus praise judgments.

### Boundary conditions

We have documented asymmetries between praise and blame in their extremity and differentiation, but it is likely that these effects might be moderated by a variety of factors. In our studies, people provided ratings about generalized targets, without having learned any individuating information about them. It is possible that our primary patterns might be attenuated, or even reversed, when perceivers have prior basis to view the target negatively—because, for example, the target is an outgroup member [7] or has a record of bad moral character [39,54]. In such cases, perceivers might assign blame more quickly than praise, and they might ramp up blame even for weak pre-action mental states, thus weakening the differentiation among them.

Reducing the anonymity of perceivers’ judgments might have divergent effects on the patterns we have revealed here. On the one hand, given that perceivers allocate more punishment when their decisions are made known to others [55], the extremity effect (whereby blame is stronger than praise) might be heightened in such public contexts. At the same time, since blame is socially costly and consequential [8], perceivers might be even more cautious when making blame decisions in public contexts, taking extra time to get it “right”. Thus, when perceivers lack anonymity when making moral judgments, they might simultaneously be more inclined to assign blame yet be slower to actually assign it.

## Conclusion

This article reported a series of studies designed to test potential asymmetries between moral judgments of blame and praise. The results revealed systematic ways in which these judgments differ. Blame tended to be more extreme than praise, even when behaviors were matched on degree of basic extremity. Blame was more differentiated: people made more fine-grained distinctions among particular mental states (i.e., thinking vs. wanting vs. intending) when assigning blame than when assigning praise. Together, these findings reflect the social costs and social regulatory function of moral judgments, suggesting that blame and praise are not mirror images and that blame might be more complex.

## Supporting information

S1 File Supporting information file contains stimuli from all studies and information about the calculation of effect sizes. (DOCX)
