# Supplementary material for: Asymmetric morality: Blame is more differentiated and more extreme than praise
Source: PLoS One. 2019 Mar 12;14(3):e0213544. doi: 10.1371/journal.pone.0213544 (PMC6413939; doi:10.1371/journal.pone.0213544)
Supplement: S1 File — (DOCX) [file pone.0213544.s001.docx]

# Supplementary Information for:

# Asymmetric Morality: Blame is More Differentiated and More Extreme than Praise

Steve Guglielmo Bertram F. Malle

Macalester College Brown University

All stimuli and data are publicly available at <https://osf.io/496sv/>.

For the proper treatment of within-subject designs, we followed the guidelines of (Dunlap, Cortina, Vaslow, & Burke, 1996). The denominator 𝜎 of Cohen’s *d* for within-subject comparisons is pooled from the standard deviations of the original means, not the smaller standard deviation of the difference score, and not the derived 𝜎 from *t* or *F* in the within-subjects ANOVA, which all benefit from the within-subject correlation and would lead to an overestimation of *d*. To calculate confidence intervals of *d* we used the variance formula for within-subject designs from (Morris, 2000, Equations 7 and 11) and the variance formula for between-subjects designs from (Morris & DeShon, 2002, p. 125, A1 and A2). When computing *d* values for interaction effects we used the formula $d= \frac{\left( a1-b1 \right)-(a2-b2)}{2\sigma}$, following the derivations by Westfall (2015).

Dunlap, W. P., Cortina, J. M., Vaslow, J. B., & Burke, M. J. (1996). Meta-analysis of experiments with matched groups or repeated measures designs. *Psychological Methods*, *1*(2), 170–177.

Morris, S. B. (2000). Distribution of the standardized mean change effect size for meta-analysis on repeated measures. *The British Journal of Mathematical and Statistical Psychology*, *53 (Pt 1)*, 17–29.

Westfall, J. (2015, October 28). Cohen’s d for 2x2 anova interaction. Retrieved October 19, 2018, from https://stats.stackexchange.com/questions/179098/cohens-d-for-2x2-anova-interaction

##### Table A

*Negative and positive behavior statements from Study 1.*

| Negative |
| --- |
| 1. smashing the rear window of a random parked car |
| 1. eating at a restaurant and walking out without paying the bill |
| 1. stealing expensive clothing from a department store |
| 1. starting a false rumor about a work colleague’s criminal past |
| 1. cheating on his/her spouse |
| Positive |
| 1. volunteering five hours per week with a non-profit organization |
| 1. participating in an effort to clean up a city park |
| 1. donating money to a charity |
| 1. offering to teach his/her teenage niece how to drive a car |
| 1. buying a hot meal for a homeless person |

##### Table B

*Average pretest ratings of social desirability for the negative and positive behaviors from Studies 2 and 3.*

| Behavior | Desirability |
| --- | --- |
| Negative |  |
| 1. set fire to his house to get insurance money for it | -4.53 |
| 1. sold drugs to high school students | -4.08 |
| 1. smashed the rear window of a random parked car | -3.78 |
| 1. started a false rumor about her ex-boyfriend | -3.34 |
| 1. harassed her neighbor’s dog | -2.66 |
| 1. parked her car illegally on a handicapped spot | -2.24 |
| 1. made a prank phone call | -1.78 |
| 1. pulled his dog’s tail | -1.59 |
|  | Avg. = -3.00 |
| Positive |  |
| 1. paid a month’s rent for a family threatened to be evicted | 4.53 |
| 1. gave out toys to the Children’s Hospital at Christmas | 4.13 |
| 1. donated money to charity | 3.72 |
| 1. bought a meal for a homeless person | 3.28 |
| 1. loaned her computer to a colleague | 2.66 |
| 1. offered to teach his/her niece how to drive a car | 2.21 |
| 1. replaced three lightbulbs in her parents’ house | 1.88 |
| 1. planted flowers around his home | 1.51 |
|  | Avg. = 2.99 |

##### Table C

*Average pretest ratings of social desirability for the negative, positive, and neutral behaviors from Study 4.*

| Sentence base | Negative | Positive | Neutral |
| --- | --- | --- | --- |
| 1. Tracy decided to [**steal from**] [*donate to*] [read about] a children's charity. | -3.39 | 3.02 | 1.83 |
| 1. While fiddling with it for a while, Frank [**broke**] [*fixed*] [wore] his friend's expensive new watch. | -2.09 | 2.46 | -.35 |
| 1. During passing time, Sally [**teased**] [*smiled at*] [noticed] the girl at the locker next to hers. | -2.17 | 2.27 | .36 |
| 1. Rhonda [**lost**] [*found*] [discussed] her friend's treasured family heirloom. | -2.24 | 2.34 | 1.15 |
| 1. The lawmaker proposed [**reducing**] [*increasing*] [evaluating] jail sentences for pedophiles. | -2.65 | 2.19 | .06 |
| 1. While at his father's house, Steve [**destroyed**] [*renovated*] [examined] his father's tool shed. | -2.85 | 2.88 | .69 |
| 1. While updating the office computer system, Dave [**deleted**] [*recovered*] [opened] several critically important files. | -2.61 | 2.27 | -.73 |
| 1. While at the museum, Tim [**damaged**] [*restored*] [contemplated] the invaluable painting. | -3.04 | 2.81 | 1.15 |
| 1. As she left for work, Mary [**taunted**] [*comforted*] [ignored] the dog barking in her neighbor's yard. | -1.93 | 2.33 | .31 |
| Average | -2.55 | 2.51 | .50 |
